# Radiotherapy utilization in the last 30 days before death among patients with malignant neoplasms in Japan: a claims database study

**DOI:** 10.1007/s10147-026-03076-1

**Published:** 2026-05-28

**Authors:** Kazuya Takeda, Rei Umezawa, Takaya Yamamoto, Noriyoshi Takahashi, Shinsaku Okuda, Keiichi Jingu

**Affiliations:** 1 Department of Radiation Oncology, Tohoku University Graduate School of Medicine, 1-1 Seiryo-machi, Aoba-ku, Sendai, 980-8574 Miyagi Japan; 2 Department of Radiation Oncology, South Miyagi Medical Center, 38-1 Nishi, Ogawara, Shibata, 989-1253 Miyagi Japan

**Keywords:** Radiotherapy, End of life, Palliative care, Claims database, Single-fraction radiotherapy, Japan

## Abstract

**Background:**

Radiotherapy (RT) utilization in the last 30 days before death has been reported at 5–10%, but data from Japan are lacking. This study aimed to characterize patterns of end-of-life RT utilization among patients with malignant neoplasms in Japan.

**Methods:**

Using the DeSC insurance claims database, patients who died between May 2016 and December 2024 with a primary malignant neoplasm diagnosis were identified. RT utilization in the last 30 days before death, treatment techniques, and the proportions of potentially excessive RT (IMRT, stereotactic irradiation, particle beam therapy, or ≥ 11 treatment days) and single-fraction RT were examined. Multivariable logistic regression was used to explore factors associated with RT utilization and single-fraction RT use.

**Results:**

Among 329,230 deceased patients, 8799 (2.7%) received RT in the last 30 days before death. Potentially excessive RT was observed in 33.7%, declining from 43.1% in 2016 to 27.2% in 2024. Single-fraction RT accounted for 5.5%, increasing from 2.8% in 2016 to 8.8% in 2024. On multivariable analysis, older age, higher comorbidity, and palliative care unit admission were associated with non-use of RT, while bone metastasis, higher-function hospitals, and urban location were associated with higher utilization. Bone metastasis, palliative care unit admission, and higher hospital function were associated with greater single-fraction RT use.

**Conclusions:**

The end-of-life RT utilization rate in Japan was 2.7%, lower than rates reported in other countries. Potentially excessive RT showed a declining trend, while single-fraction RT showed an increasing trend. These findings provide baseline data for optimizing end-of-life RT practices in Japan.

## Introduction

In the end-of-life care of patients with malignant neoplasms, providing appropriate supportive care while avoiding overly aggressive treatment is important for both patient quality of life and healthcare economics. Radiotherapy (RT) is a widely used treatment modality in both curative and palliative settings for patients with malignant neoplasms. The utilization rate of RT in the last 30 days before death has been reported at 5–10% [1–6]. Because end-of-life RT encompasses both overtreatment and necessary palliative care, defining an optimal utilization rate is inherently difficult [2]. Nonetheless, characterizing this rate and the details of RT delivered near the end of life is essential for optimizing RT use.

In Japan, overall RT utilization among patients with malignant neoplasms is lower than in other countries [7–9], yet the rate of RT use in the last 30 days before death remains unknown. This study therefore aimed to investigate patterns of RT utilization in the last 30 days before death using a Japanese insurance claims database.

## Patients and methods

### Data source

This study used the DeSC database (DeSC Healthcare, Tokyo, Japan), a commercial claims database in Japan. Under Japan’s universal health insurance system, all citizens must enroll in a public health insurance plan. The DeSC database aggregates data from multiple insurers, including Health Insurance Societies covering employees and their dependents, National Health Insurance covering primarily self-employed individuals and retirees, and the Latter-Stage Elderly Healthcare System primarily covering individuals aged 75 years or older. By analyzing claims data from these insurers, the database enables tracking of all insured medical services and drug prescriptions across a broad age range. For this study, a dataset spanning April 2014 to February 2025 was obtained on September 5, 2025.

### Study population

Death events and dates were defined as follows. Deceased patients were identified by meeting at least one of the following criteria: (1) a positive death flag in the patient master information; (2) insurance eligibility loss with the reason recorded as death; (3) an inpatient diagnosis outcome recorded as death; or (4) billing of procedure codes specific to the time of death. The date of death was determined as the last date of procedure billing within the estimated month of death. Among patients with an identified date of death, those who died during the study period (May 2016 to December 2024) and whose principal diagnosis in the last billing month was a primary malignant neoplasm were included in the analysis cohort. The study period began in May 2016 because RT administered during palliative care unit stays was not captured in claims data through March 2016. In addition, the presence of a bone metastasis diagnosis (ICD-10 code C79.5) within the last 30 days before death was assessed from claims for the month of death and the preceding month, because diagnoses in claims data are recorded on a monthly basis.

### Study variables

In addition to the primary cancer site and bone metastasis diagnosis described above, data on sex, age at death, and date of death were collected. Urban classification was assigned based on Tokyo’s special wards, government-designated cities, and core cities determined by the mode of billing facility locations during the last 30 days before death. Hospital function was assessed from all billing records in the last 30 days before death, and when multiple facilities were involved, the highest-ranked hospital was assigned. Comorbidity burden was evaluated using the Charlson Comorbidity Index (CCI) [10]. Detection and scoring of comorbidities from insurance billing codes followed the 2011 revised algorithm of Quan et al. [11]. Although the original CCI assigns specific weights to a malignancy diagnosis and to metastatic disease, these conditions were systematically excluded from score calculation in this study because the cohort consisted entirely of terminal patients with malignant neoplasms. This cancer-specific CCI excluding these diagnoses was adopted in line with previous claims-based reports [12, 13]. The total CCI was calculated from diagnoses recorded during the 12 months before the month of death and categorized as 0, 1, 2, or ≥ 3. Billing for chemotherapy drug costs and palliative care unit admission fees in the last 30 days before death were examined as surrogate indicators of active cancer treatment and advanced palliative care utilization, respectively.

### Radiotherapy (RT)

Consecutive claims with intervals of fewer than 14 days were grouped as a single RT course. For three-dimensional conformal radiotherapy (3D-CRT) and intensity-modulated radiotherapy (IMRT), billing was generated per treatment day, allowing calculation of the number of treatment days. RT was considered utilized if the end date of the course was within the last 30 days before death. Among qualifying courses, the one closest to death was designated as the patient’s last RT course, and treatment techniques and number of treatment days were tabulated. The intervals from the start and end dates of the last RT course to death were also calculated.

Potentially excessive RT was defined as a last RT course meeting at least one of the following criteria: (1) use of IMRT, stereotactic irradiation, or particle beam therapy; or (2) 11 or more treatment days. Conversely, single-fraction RT can be considered an appropriate approach in the end-of-life setting because it provides equivalent pain relief to fractionated regimens for bone metastases [14]. In Japan, single-fraction RT in palliative settings is most commonly billed under the code M001-3, which was therefore used as an operational definition of single-fraction RT in this study. Both potentially excessive and single-fraction RT courses were tabulated separately.

### Statistical analysis

Values were reported as counts with proportions or medians with interquartile ranges (IQRs), as appropriate. Among patients who received RT in the last 30 days before death, the distribution of treatment techniques, proportion of potentially excessive RT, and utilization rate of single-fraction RT were calculated. Intervals from the start and end of the last RT course to death were presented as medians with IQRs. To explore factors associated with RT utilization in the last 30 days before death, multivariable logistic regression was performed. Continuous variables (age, date of death) were modeled using restricted cubic splines (RCS, 4 knots) to capture potential nonlinear relationships. For each continuous variable, the overall association with the outcome was tested by a joint Wald test of all spline terms (overall p value), and nonlinearity was tested by a joint Wald test of the nonlinear terms only (p for nonlinearity). Each association was visualized using spline curves of adjusted Odds ratios (ORs) with 95% confidence intervals plotted relative to a reference value. ORs for continuous variables represented the effect of a change from the 25th to the 75th percentile. A similar RCS multivariable logistic regression was performed in the subgroup receiving RT in the last 30 days, examining factors associated with single-fraction RT use in the last RT course. Both models included chemotherapy use and palliative care unit admission in the last 30 days as covariates. All p-values were evaluated at a two-sided significance level of 5%. As this study was descriptive and exploratory, no adjustment for multiplicity was performed.

Data extraction and processing were performed using Python version 3.9.6 (Python Software Foundation, Wilmington, DE, USA) with the DuckDB library version 1.4.1 (DuckDB Labs, Amsterdam, Netherlands). Statistical analyses were conducted using R version 4.5.1 (R Foundation for Statistical Computing, Vienna, Austria).

### Ethical considerations

This observational study used an anonymized database, and informed consent was waived. The study was approved by the Ethics Committee of Tohoku University Graduate School of Medicine (approval number: 2025-1-452).

### Use of generative AI

Generative AI-related tools, including ChatGPT, Claude, and Cursor, were used in this study for drafting analysis code and assisting with manuscript preparation. The final analysis code and manuscript were reviewed, revised, and finalized under the responsibility of the authors.

## Results

### Identification of deceased patients and patient characteristics

Of 2,176,045 patients with a primary malignant neoplasm diagnosis in the dataset, death flag information was available for 1,858,163 (85.4%), yielding 539,527 deaths. Combining these with deaths detected by other criteria resulted in a total of 685,064 deceased patients. Of these, 588,584 had an identified date of death within the study period (May 2016 to December 2024), and 329,230 whose principal diagnosis in the last billing month was a primary malignant neoplasm were included in the analysis.

Table 1 shows patient characteristics for the overall cohort and subgroups stratified by RT use. The median age of patients who received RT was 76.2 years, and lung cancer was the most common primary site (41.0%). Among patients who received RT, 7.6% received chemotherapy in the last 30 days before death and 23.4% had palliative care unit admission billing.

**Table 1:** Patient characteristics

Variable	Overall (*N* = 329,230)	No RT (*N* = 320,431)	RT (*N* = 8,799)	SMD
Age at death (years), median [IQR]	81.0 [74.8–86.8]	81.1 [74.9–86.9]	76.2 [70.3–81.5]	0.54
Female, n (%)	131,660 (40.0)	129,007 (40.3)	2653 (30.2)	0.21
Death year, n (%)				0.13
2016	10,082 (3.1)	9722 (3.0)	360 (4.1)	
2017	18,237 (5.5)	17,629 (5.5)	608 (6.9)	
2018	35,655 (10.8)	34,595 (10.8)	1060 (12.0)	
2019	46,710 (14.2)	45,379 (14.2)	1331 (15.1)	
2020	52,741 (16.0)	51,349 (16.0)	1392 (15.8)	
2021	55,444 (16.8)	53,984 (16.8)	1460 (16.6)	
2022	54,427 (16.5)	53,064 (16.6)	1363 (15.5)	
2023	43,515 (13.2)	42,529 (13.3)	986 (11.2)	
2024	12,419 (3.8)	12,180 (3.8)	239 (2.7)	
Primary cancer site, n (%)				0.67
Head and neck	7563 (2.3)	7230 (2.3)	333 (3.8)	
Esophagus	9315 (2.8)	8849 (2.8)	466 (5.3)	
Stomach	37,820 (11.5)	37,404 (11.7)	416 (4.7)	
Colon/rectum	43,343 (13.2)	42,805 (13.4)	538 (6.1)	
Liver	20,305 (6.2)	20,014 (6.2)	291 (3.3)	
Pancreas	29,677 (9.0)	29,398 (9.2)	279 (3.2)	
Lung	61,440 (18.7)	57,831 (18.0)	3609 (41.0)	
Breast	11,186 (3.4)	10,881 (3.4)	305 (3.5)	
Uterus/ovary	8012 (2.4)	7788 (2.4)	224 (2.5)	
Prostate	15,069 (4.6)	14,735 (4.6)	334 (3.8)	
Blood	24,131 (7.3)	23,606 (7.4)	525 (6.0)	
Others	47,813 (14.5)	46,674 (14.6)	1139 (12.9)	
Multiple	13,556 (4.1)	13,216 (4.1)	340 (3.9)	
*Bone metastasis*,* n (%)*	*42*,*407 (12.9)*	*37*,*686 (11.8)*	*4*,*721 (53.7)*	*1.00*
Hospital function, n (%)				1.15
University	20,247 (6.1)	18,610 (5.8)	1637 (18.6)	
DPC advanced	54,087 (16.4)	51,051 (15.9)	3036 (34.5)	
DPC standard	118,368 (36.0)	114,477 (35.7)	3891 (44.2)	
Non-DPC	114,342 (34.7)	114,130 (35.6)	212 (2.4)	
Unknown	22,186 (6.7)	22,163 (6.9)	23 (0.3)	
Urban, n (%)				0.19
Suburban	108,219 (32.9)	105,703 (33.0)	2516 (28.6)	
Urban	148,260 (45.0)	143,492 (44.8)	4768 (54.2)	
Unknown	72,751 (22.1)	71,236 (22.2)	1515 (17.2)	
CCI, n (%)				0.24
0	100,528 (30.5)	97,323 (30.4)	3205 (36.4)	
1	39,918 (12.1)	38,392 (12.0)	1526 (17.3)	
2	67,494 (20.5)	65,955 (20.6)	1539 (17.5)	
*≥*3	121,290 (36.8)	118,761 (37.1)	2529 (28.7)	
Chemotherapy in the last 30 days, n (%)	12,940 (3.9)	12,269 (3.8)	671 (7.6)	0.16
Palliative care unit in the last 30 days, n (%)	61,537 (18.7)	59,476 (18.6)	2061 (23.4)	0.12

CCI Charlson comorbidity index, DPC diagnosis procedure combination, IQR interquartile range, RT radiotherapy, SMD standardized mean difference

### RT utilization in the last 30 days before death

Overall, 8,799 patients (2.7%) had RT billing in the last 30 days before death. Stratified by hospital function, the RT utilization rate in the last 30 days before death was 8.1% in patients in the university hospital group, 5.6% in those in DPC advanced hospitals, 3.3% in DPC standard hospitals, and 0.2% in non-DPC hospitals. In the last RT course, 3D-CRT was billed in the majority of cases (85.9%), and single-fraction RT accounted for 5.5%. IMRT was billed in 5.6%, and special irradiation techniques including stereotactic irradiation and particle beam therapy were billed in 6.2% (the total exceeds 100% because multiple techniques could be billed within a single course). Among the 7,964 patients whose last RT course consisted of 3D-CRT or IMRT, 32.3% received RT over 5 or fewer treatment days, 38.9% over 6 to 10 treatment days, and 28.8% over 11 or more treatment days, respectively. The median interval from the start of the last RT course to death was 25 days (IQR: 15–35), and that from the end of the last course to death was 13 days (IQR: 6–21).

### Factors associated with RT use in the last 30 days before death and single-fraction RT use

**Fig. 1:**
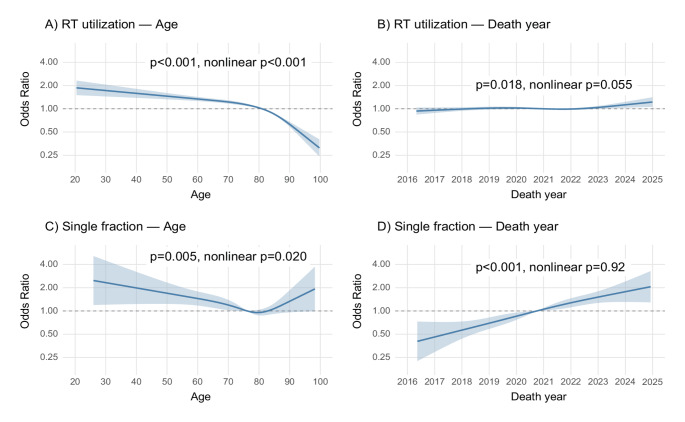
Adjusted odds ratios for end-of-life radiotherapy utilization by age and date of death. (**A**) Age and RT utilization in the last 30 days before death. (**B**) Date of death and RT utilization in the last 30 days before death. (**C**) Age and single-fraction RT use among patients with RT in the last 30 days. (**D**) Date of death and single-fraction RT use among patients with RT in the last 30 days. Solid lines indicate adjusted ORs; shaded areas indicate 95% confidence intervals. The reference value (OR = 1) is set at the median of each continuous variable. All models used restricted cubic spline logistic regression with 4 knots (placed at the 5th, 35th, 65th, and 95th percentiles). OR, odds ratio; RT, radiotherapy

Factors associated with RT utilization in the last 30 days before death were examined using multivariable logistic regression (Table 2). Age showed a significant nonlinear association (overall *p* < 0.001, nonlinear *p* < 0.001), with utilization decreasing with advancing age, particularly after 80 years (Fig. 1a). Date of death was significantly associated with RT use (*p* = 0.018), but nonlinearity was borderline (*p* = 0.055; Fig. 1b). The presence of a bone metastasis diagnosis was strongly associated with RT use (OR 6.38, 95% CI 6.07–6.69, *p* < 0.001). Hospital function was also significantly associated with RT use (*p* < 0.001). With DPC standard hospitals as the reference, the OR was 2.11 (95% CI 1.98–2.25) for university hospitals and 1.57 (95% CI 1.49–1.65) for DPC advanced hospitals, whereas the OR for non-DPC hospitals was 0.06 (95% CI 0.06–0.07). Among the remaining factors, higher CCI scores and palliative care unit admission were associated with non-use of RT, whereas male sex, and urban location were associated with higher utilization. Significant differences by primary site were also observed. Chemotherapy use did not show a significant association (*p* = 0.142).

**Table 2:** Factors associated with RT use in the last 30 days before death (*N* = 329,230)

Variable	OR (95% CI)	*P*-value
Age (IQR: 74.8 to 86.8 years)	0.67 (0.63–0.71)	*< 0.001*
Death date (IQR: 2019.4 to 2022.5)	0.99 (0.93–1.05)	*0.018*
Female (ref: Male)	0.83 (0.79–0.88)	*< 0.001*
Primary cancer site (ref: Colon/rectum)		*< 0.001*
Head and neck	2.91 (2.51–3.36)	
Esophagus	3.00 (2.63–3.42)	
Stomach	0.92 (0.81–1.05)	
Liver	1.02 (0.88–1.18)	
Pancreas	0.72 (0.62–0.84)	
Lung	2.81 (2.56–3.10)	
Breast	0.87 (0.75–1.02)	
Uterus/ovary	1.80 (1.52–2.13)	
Prostate	0.76 (0.66–0.88)	
Blood	1.58 (1.39–1.79)	
Others	1.55 (1.40–1.73)	
Multiple	1.38 (1.20–1.59)	
*Bone metastasis*	6.38 (6.07–6.69)	*< 0.001*
Hospital function (ref: *DPC standard*)		*< 0.001*
University	2.11 (1.98–2.25)	
DPC advanced	1.57 (1.49–1.65)	
*Non-DPC*	0.06 (0.06–0.07)	
Unknown	0.04 (0.03–0.07)	
Urban (ref: Suburban)		*< 0.001*
Urban	1.25 (1.19–1.32)	
Unknown	1.20 (1.12–1.29)	
CCI (ref: 0)		*< 0.001*
1	0.93 (0.87–1.00)	
2	0.86 (0.80–0.92)	
*≥*3	0.82 (0.77–0.87)	
Palliative care unit in the last 30 days	0.77 (0.73–0.81)	*< 0.001*
Chemotherapy in the last 30 days	0.94 (0.86–1.02)	*0.142*

CI confidence interval, CCI Charlson comorbidity index, DPC diagnosis procedure combination, IQR interquartile range, OR odds ratio, RCS restricted cubic splines, RT radiotherapy. Continuous variables (age, death date) were modeled using RCS with 4 knots; ORs represent the effect of change from the 25th to the 75th percentile. *P*-values for continuous variables are from the overall Wald test. The age effect showed significant nonlinearity (*p* < 0.001); the death date effect did not (*p* = *0.055*)

Among the 8799 patients with RT billing in the last 30 days before death, factors associated with single-fraction RT in the last RT course were explored for the 488 patients (5.5%) who received single-fraction RT (Table 3). Age showed a significant nonlinear association (overall *p* = 0.005, nonlinear *p*  = 0.020; Fig. 1c). Date of death was significantly associated with increased single-fraction RT use in an approximately linear fashion (OR for IQR change: 1.88, 95% CI 1.43–2.47, nonlinear *p* = 0.92; Fig. 1d). The presence of a bone metastasis diagnosis was significantly associated with higher single-fraction RT use (OR 3.48, 95% CI 2.76–4.39, *p* < 0.001), and palliative care unit admission was significantly associated with higher single-fraction RT utilization (OR 1.55, *p* < 0.001). Hospital function (*p*  = 0.002), facility location (*p* = 0.027), and primary site (*p* = 0.047) were also significant. Chemotherapy use showed a borderline association (OR 0.68, *p* = 0.076).

**Table 3:** Factors associated with single-fraction RT in the last RT course among patients with RT in the last 30 days (*N* = 8799)

Variable	OR (95% CI)	*P*-value
Age (IQR: 70.3 to 81.5 years)	0.82 (0.65–1.04)	*0.005*
Death date (IQR: 2019.1 to 2022.3)	1.88 (1.43–2.47)	*< 0.001*
Female (ref: Male)	1.03 (0.83–1.29)	*0.792*
Primary cancer site (ref: Colon/rectum)		*0.047*
Head and neck	1.04 (0.59–1.86)	
Esophagus	0.80 (0.45–1.43)	
Stomach	0.80 (0.46–1.39)	
Liver	0.80 (0.44–1.46)	
Pancreas	1.38 (0.82–2.34)	
Lung	0.73 (0.51–1.06)	
Breast	0.43 (0.22–0.86)	
Uterus/ovary	0.62 (0.29–1.34)	
Prostate	0.78 (0.45–1.36)	
Blood	1.04 (0.59–1.83)	
Others	0.79 (0.51–1.20)	
Multiple	1.23 (0.74–2.04)	
*Bone metastasis*	3.48 (2.76–4.39)	*< 0.001*
Hospital function (ref: *DPC standard*)		*0.002*
University	1.59 (1.23–2.06)	
DPC advanced	1.41 (1.14–1.75)	
*Non-DPC*	1.43 (0.79–2.59)	
Unknown	2.72 (0.61–12.05)	
Urban (ref: Suburban)		*0.027*
Urban	1.19 (0.96–1.49)	
Unknown	0.84 (0.62–1.14)	
CCI (ref: 0)		*0.943*
1	0.92 (0.70–1.22)	
2	0.97 (0.74–1.27)	
*≥*3	0.99 (0.78–1.26)	
Palliative care unit in the last 30 days	1.55 (1.27–1.90)	*< 0.001*
Chemotherapy in the last 30 days	0.68 (0.44–1.04)	*0.076*

CI confidence interval, CCI Charlson comorbidity index, DPC diagnosis procedure combination, IQR interquartile range, OR odds ratio, RCS restricted cubic splines, RT radiotherapy. Continuous variables (age, death date) were modeled using RCS with 4 knots; ORs represent the effect of change from the 25th to the 75th percentile. *P*-values for continuous variables are from the overall Wald test. The death date effect was approximately linear (nonlinear *p* = *0.92*); the age effect showed borderline nonlinearity (nonlinear *p* = *0.020*)

### Patterns of potentially excessive RT and single-fraction RT

Among the 8,799 patients who received RT in the last 30 days before death, IMRT or special irradiation techniques were used in 11.8%, and 11 or more treatment days were recorded in 26.0%. Overall, 33.7% met at least one criterion for potentially excessive RT, and this proportion declined from 43.1% in 2016 to 27.2% in 2024. Conversely, single-fraction RT accounted for 5.5% of last RT courses, with utilization increasing from 2.8% in 2016 to 8.8% in 2024 (Table 4).

**Table 4:** Proportion of potentially excessive RT and of single-fraction RT by death year among patients with RT in the last 30 days before death

Death year	*N* (RT users)	Potentially excessive RT, *n* (%)	Single fraction RT, *n* (%)
2016	360	155 (43.1)	10 (2.8)
2017	608	254 (41.8)	15 (2.5)
2018	1060	399 (37.6)	52 (4.9)
2019	1331	470 (35.3)	50 (3.8)
2020	1392	453 (32.5)	69 (5.0)
2021	1460	448 (30.7)	87 (6.0)
2022	1363	419 (30.7)	101 (7.4)
2023	986	302 (30.6)	83 (8.4)
2024	239	65 (27.2)	21 (8.8)

Potentially excessive RT: last RT course met ≥ 1 of (i) IMRT or special irradiation, (ii) ≥ 11 treatment days. Single fraction: last RT course was single-fraction

## Discussion

This study investigated patterns of RT utilization in the last 30 days before death among patients with malignant neoplasms in Japan using an insurance claims database. Although studies from other countries have reported on end-of-life RT utilization [1–6], previous data from Japan have been limited to a report on in-hospital deaths [15]. To our knowledge, this is the first comprehensive analysis of RT utilization before death among patients with malignant neoplasms in Japan, filling an important gap in understanding end-of-life care practices.

First, this study employed multiple algorithms to maximize identification of deceased patients. In Japanese insurance claims data, death is not directly recorded in claims, and is instead estimated from DPC discharge records and death-specific procedure codes. This approach has been noted to yield insufficient death identification rates, particularly for outpatients [16, 17]. However, in the DeSC database, insurer-held death information was available for 85.4% of the analyzed population, and by combining this with billing code–based death estimation, a relatively high identification rate was achieved, including outpatient deaths.

The overall RT utilization rate in the last 30 days before death has been reported as 5–10% in a systematic review of studies from other countries than Japan [1]. Sato et al. reported an RT utilization rate of 12.7% in the last 30 days before death among patients who died at a high-volume center in Japan [15]. In our study, the overall RT utilization rate was 2.7%, lower than these previously reported rates. Even within the university hospitals, which accounted for 6.1% of the cohort, the rate was only 8.1%. In addition to a low end-of-life RT utilization rate in Japan as a whole, these findings indicate substantial disparities in RT access across hospital categories. Japan has long been noted to have lower overall RT utilization among cancer patients compared with other countries [7–9], and this disparity appears to extend to the end-of-life setting. Japan’s RT infrastructure is fragmented across many small facilities each operating only a few accelerators, although per-capita number of linear accelerators is comparable to that of other developed countries [18]. Moreover, more than 99% of the population can reach an RT facility within a 60-minute drive [19]. The persistently low end-of-life RT utilization despite this favorable geographic access suggests that referral to nearby RT-equipped facilities remains insufficient, leaving some patients with malignant neoplasms without access to necessary palliative RT. Among patients who received RT in the last 30 days before death, the single-fraction RT rate was 5.5%. A previous systematic review reported wide international variation (0–59%) [1], and the value observed in Japan was relatively low within this range.

On multivariable analysis, older age, higher CCI scores, and palliative care unit admission were associated with non-use of RT, while male sex, higher-function hospitals, and urban location were associated with higher utilization, consistent with previous reports [2, 5]. In addition, the presence of a bone metastasis diagnosis was strongly associated with RT use in our cohort (OR 6.38, *p* < 0.001). Although a bone metastasis diagnosis code does not necessarily indicate that RT was directed at the metastasis, this diagnosis was identified in 53.7% of RT recipients compared with 11.8% of those without RT, suggesting that RT for bone metastasis accounts for a substantial portion of end-of-life RT use. Bone metastasis was also significantly associated with single-fraction RT, consistent with the well-established evidence for single-fraction regimens (such as 8 Gy in a single fraction) in the palliation of painful bone metastases [14]. Nonlinear modeling of age revealed a particularly steep decline in utilization after age 80 (overall *p* < 0.001, nonlinear *p* < 0.001), suggesting potentially insufficient RT access among elderly patients. For single-fraction RT, age showed a nonlinear U-shaped association (overall *p*  = 0.005, nonlinear *p* = 0.020), with utilization lowest around age 80 and higher in both younger and very elderly patients. Among younger patients, this may reflect continued treatment interventions near the end of life; among very elderly patients, it may reflect a preference for single-fraction RT to minimize treatment burden. Date of death was significant for both overall RT utilization (*p* = 0.018) and single-fraction RT utilization (*p* < 0.001), with a nearly linear increasing trend particularly for single-fraction RT, suggesting gradual adoption over time. Primary site was also associated with both outcomes, likely reflecting disease-specific characteristics such as natural history and patterns of metastatic spread.

Single-fraction RT is considered an appropriate palliative modality, particularly for pain relief [14]. Comparing overall RT and single-fraction RT utilization patterns can therefore provide insights into appropriate palliative RT use. Overall RT utilization was significantly lower among patients with palliative care unit admissions, while single-fraction RT utilization was significantly higher in this same group. This suggests that facilities providing advanced palliative care may make more appropriate decisions regarding both avoidance of excessive treatment and use of palliative RT. RT utilization was markedly higher at university and DPC hospitals than at non-DPC hospitals (non-DPC hospitals vs. DPC standard hospitals: OR 0.06, 95% CI 0.06–0.07), likely because most RT-equipped facilities are DPC hospitals. Among DPC hospitals, university and advanced DPC hospitals showed higher overall RT utilization in the last 30 days before death, probably reflecting the dual curative and palliative role of these higher-function institutions. Hospital function was also significant in the single-fraction RT analysis (*p* = 0.002), suggesting that appropriate single-fraction RT use may be more prevalent at hospitals with advanced capabilities. Taken together, higher hospital function and the presence of specialized palliative care appear to promote single-fraction RT selection in current Japanese practice. The increase in single-fraction RT from 2.8% in 2016 to 8.8% in 2024 suggests that collaboration between RT and palliative care departments has the potential to promote appropriate palliative RT use.

Although accurately determining overtreatment among patients who received RT in the last 30 days before death was difficult, several reference values were derived. Use of IMRT, stereotactic, or special irradiation techniques was observed in 11.8%, and 11 or more treatment days were recorded in 26.0%, with 33.7% meeting at least one criterion for potentially excessive RT. In Japan, some IMRT cases are billed as 3D-CRT at facilities that do not meet the IMRT billing requirements. The use of IMRT techniques in our cohort may therefore be slightly underestimated. While several models have been developed to predict 1-month survival in patients with advanced malignant neoplasms, their accuracy remains limited [20–22], and rapid deterioration during an ongoing course can occur even in non-terminal patients. Therefore, not all these cases necessarily represent inappropriate treatment decisions. Nevertheless, these indicators may serve as benchmarks for future efforts to optimize RT utilization. The proportion of single-fraction RT (5.5%) may similarly serve as a reference for promoting appropriate palliative RT. Temporal trends showed a decrease in potentially excessive RT alongside an increase in single-fraction RT, suggesting a gradual shift toward more appropriate palliative RT use over time.

This study has several limitations. First, the clinical intent (curative or palliative) of individual RT courses could not be identified, precluding accurate distinction between overtreatment or unexpected death during non-palliative treatment and appropriate end-of-life palliative care. Second, owing to the characteristics of the DeSC database, patient population composition was not entirely consistent across calendar years, and the representativeness of the study cohort has certain limitations. Third, single-fraction RT was operationally defined by the M001-3 billing code. However, some single-fraction RT may be billed under other codes, and some non-single-fraction RT may also be billed under M001-3. Similarly, IMRT performed under the 3D-CRT billing code at some facilities could not be identified. The sensitivity and specificity of these claims-based variable definitions were not validated in our study, and further investigation is warranted.

In conclusion, this study characterized patterns of RT utilization in the last 30 days before death among patients with malignant neoplasms in Japan. The RT utilization rate was 2.7%, lower than rates reported in other countries, suggesting potential underutilization of palliative RT. Potentially excessive treatment was observed in 33.7% of last RT courses with a declining trend, while single-fraction RT (5.5%) showed an increasing trend. These findings provide baseline data for optimizing end-of-life RT practices in Japan.

## Data Availability

The data that support the findings of this study are available from DeSC Healthcare but restrictions apply to the availability of these data, which were used under license for the current study, and so are not publicly available.
